# 620 Communication in a Flash: Evolution of Nurse-led Newsletters in a Burn Center

**DOI:** 10.1093/jbcr/iraf019.249

**Published:** 2025-04-01

**Authors:** Stacey Richerbach, Tiffany Hockenberry, Karen Richey, Kevin Foster

**Affiliations:** Diane & Bruce Halle Arizona Burn Center; Diane & Bruce Halle Arizona Burn Center; Diane & Bruce Halle Arizona Burn Center; Diane & Bruce Halle Arizona Burn Center

## Abstract

**Introduction:**

Newsletters, a common method of standardizing communication, are aimed at engaging and informing teams, yet often fall short of their objectives. Historically, our center distributed a monthly newsletter, and staff were accountable for reading and incorporating content into their practice. Despite expectations, it was common for individuals to report they had not been informed. Recognizing that communication vital to cultivate integrated knowledge was limited by space and time, the newsletter was rebranded to the Friday Flash with weekly distribution. Only modest improvements were noted. In 2020, nurse leaders reconceptualized the newsletter’s function and design to expand readership and optimize communication. The purpose of this study was to evaluate efficacy of these changes.

**Methods:**

The ‘Flash’ was redesigned for ease of weekly manipulation, via six-page template amenable to varying themes and color schemes. Weekly content was solicited from team leaders. A 28-item anonymous survey with positive (POS), neutral, and negatively phrased questions was distributed via Forms. Questions were categorized into four groups: informational, performance, outreach/support and recognition. Descriptive statistics were performed.

**Results:**

Forty-six team members responded. A majority (89.1%) read the Flash each week via email. Aesthetic variation in themes and color had the greatest POS association (97.8%), followed by variety of topics (95%). Most (97.9%) felt that it was a valuable communication tool and it performed well providing interdisciplinary knowledge (93.5%). Respondents indicated content was informative (100%), and 60.9% refer to prior editions of the Flash to refresh knowledge. Content was rated by interest level. Topics of greatest interest were informational (82.8%). The most favored section was “Hot Topics for Huddle” (95.7%), then education opportunities (93.5%), peer recognition (93.5%), patient feedback (89.1%), quality improvement (87%), volunteer opportunities (80.4%), cover stories (80%), clinical documentation for providers (77.4%), research updates (76.1%), Benchmark Briefs (76.9%), patient experience (69.6%), pharmacy-related info (69.6%), employee resources (67.4%), and Charting Champions (41.3%).

**Conclusions:**

Nurse leaders successfully enhanced the newsletter. Interest and readership were met with positive feedback, indicating value as a resource and mode for interdisciplinary communication. In our center ‘Flash’ has become more than a mechanism of burn injury; it is synonymous with communication.

**Applicability of Research to Practice:**

Communication amongst burn professionals is critical to an interdisciplinary approach to patient care. This project may serve as a framework for nurse-led enhancement of communication.

**Funding for the Study:**

N/A

